# Understanding the burden of interstitial lung disease post-COVID-19: the UK Interstitial Lung Disease-Long COVID Study (UKILD-Long COVID)

**DOI:** 10.1136/bmjresp-2021-001049

**Published:** 2021-09-23

**Authors:** Jim M Wild, Joanna C Porter, Philip L Molyneaux, Peter M George, Iain Stewart, Richard James Allen, Raminder Aul, John Kenneth Baillie, Shaney L Barratt, Paul Beirne, Stephen M Bianchi, John F Blaikley, Jonathan Brooke, Nazia Chaudhuri, Guilhem Collier, Emma K Denneny, Annemarie Docherty, Laura Fabbri, Michael A Gibbons, Fergus V Gleeson, Bibek Gooptu, Ian P Hall, Neil A Hanley, Melissa Heightman, Toby E Hillman, Simon R Johnson, Mark G Jones, Fasihul Khan, Rod Lawson, Puja Mehta, Jane A Mitchell, Manuela Platé, Krisnah Poinasamy, Jennifer K Quint, Pilar Rivera-Ortega, Malcolm Semple, A John Simpson, DJF Smith, Mark Spears, LIsa G Spencer, Stefan C Stanel, David R Thickett, A A Roger Thompson, Simon LF Walsh, Nicholas D Weatherley, Mark Everard Weeks, Dan G Wootton, Chris E Brightling, Rachel C Chambers, Ling-Pei Ho, Joseph Jacob, Karen Piper Hanley, Louise V Wain, R Gisli Jenkins

**Affiliations:** 1 Department of Infection, Immunity and Cardiovascular Disease, The University of Sheffield, Sheffield, UK; 2 Centre for Inflammation and Tissue Repair, UCL Respiratory, University College London, London, UK; 3 Respiratory Medicine, University College London Hospitals NHS Foundation Trust, London, UK; 4 Department of Respiratory Medicine, University College London, London, UK; 5 National Heart and Lung Institute, Imperial College London, London, UK; 6 Department of Interstitial Lung Disease, Royal Brompton and Harefield Hospital, Guys and St Thomas' NHS Foundation Trust, London, UK; 7 Department of Health Sciences, University of Leicester, Leicester, UK; 8 Respiratory Medicine, St George's Hospital NHS Foundation Trust, London, UK; 9 Roslin Institute, University of Edinburgh, Midlothian, UK; 10 Bristol Interstitial Lung Diseases Service, North Bristol NHS Trust, Bristol, UK; 11 Respiratory Medicine, Leeds Teaching Hospitals NHS Trust, Leeds, UK; 12 Academic Department of Respiratory Medicine, Sheffield Teaching Hospitals NHS Foundation Trust, Sheffield, UK; 13 Manchester Academic Health Science Centre, Manchester University NHS Foundation Trust, Manchester, UK; 14 Faculty of Biology, Medicine and Health, The University of Manchester, Manchester, UK; 15 Department of Respiratory Medicine, Nottingham University Hospitals NHS Trust, Nottingham, UK; 16 NIHR Nottingham Biomedical Research Centre, University of Nottingham, Nottingham, UK; 17 School of Medicine, University of Nottingham, Nottingham, UK; 18 Respiratory Department, University Hospital of South Manchester NHS Foundation Trust, Manchester, UK; 19 Centre for Medical Informatics, The Usher Institute, University of Edinburgh, Edinburgh, UK; 20 Respiratory Medicine, Royal Devon and Exeter NHS Foundation Trust, Exeter, UK; 21 College of Medicine and Health, University of Exeter, Exeter, UK; 22 Department of Oncology, University of Oxford, Oxford, UK; 23 Department of Molecular and Cell Biology, University of Leicester, Leicester, UK; 24 Institute for Lung Health, Leicester NIHR Biomedical Research Centre, University of Leicester, Leicester, UK; 25 Wythenshaw Hospital, Manchester University NHS Foundation Trust, Manchester, UK; 26 Clinical and Experimental Sciences, Faculty of Medicine, University of Southampton, Southampton, UK; 27 Southampton NIHR Biomedical Research Centre, University Hospital Southampton, Southampton, UK; 28 School of Life & Medical Sciences, UCL, London, UK; 29 UCL Respiratory, UCL, London, UK; 30 Head of Research and Innovation Advocacy, Asthma UK, London, UK; 31 Child Health, University of Liverpool, Liverpool, UK; 32 Translational and Clinical Research Institute, Newcastle University, Newcastle upon Tyne, UK; 33 Newcastle upon Tyne Hospitals NHS Foundation Trust, Newcastle upon Tyne, UK; 34 Respiratory Medicine, Perth Royal Infirmary, NHS Tayside, Perth, UK; 35 School of Medicine, University of Dundee, Dundee, UK; 36 Respiratory Medicine, Liverpool University Hospitals NHS Foundation Trust, Liverpool, UK; 37 Division of Diabetes, Endocrinology & Gastroenterology, The University of Manchester, Manchester, UK; 38 Birmingham Acute Care Research Group, University of Birmingham, Birmingham, UK; 39 Acute and Respiratory Medicine, University Hospitals Birmingham Foundation Trust, Birmingham, uk; 40 Institute of Infection Veterinary and Ecological Science, University of Liverpool, Liverpool, UK; 41 MRC Human Immunology Unit, Weatherall Institute of Molecular Medicine Oncology, Oxford, UK; 42 Oxford Centre for Respiratory Medicine, Churchill Hospital, Oxford, UK; 43 Centre for Medical Imaging and Computing, University College London, London, UK

**Keywords:** COVID-19, interstitial fibrosis, bronchoscopy

## Abstract

**Introduction:**

The COVID-19 pandemic has led to over 100 million cases worldwide. The UK has had over 4 million cases, 400 000 hospital admissions and 100 000 deaths. Many patients with COVID-19 suffer long-term symptoms, predominantly breathlessness and fatigue whether hospitalised or not. Early data suggest potentially severe long-term consequence of COVID-19 is development of long COVID-19-related interstitial lung disease (LC-ILD).

**Methods and analysis:**

The UK Interstitial Lung Disease Consortium (UKILD) will undertake longitudinal observational studies of patients with suspected ILD following COVID-19. The primary objective is to determine ILD prevalence at 12 months following infection and whether clinically severe infection correlates with severity of ILD. Secondary objectives will determine the clinical, genetic, epigenetic and biochemical factors that determine the trajectory of recovery or progression of ILD. Data will be obtained through linkage to the Post-Hospitalisation COVID platform study and community studies. Additional substudies will conduct deep phenotyping. The Xenon MRI investigation of Alveolar dysfunction Substudy will conduct longitudinal xenon alveolar gas transfer and proton perfusion MRI. The POST COVID-19 interstitial lung DiseasE substudy will conduct clinically indicated bronchoalveolar lavage with matched whole blood sampling. Assessments include exploratory single cell RNA and lung microbiomics analysis, gene expression and epigenetic assessment.

**Ethics and dissemination:**

All contributing studies have been granted appropriate ethical approvals. Results from this study will be disseminated through peer-reviewed journals.

**Conclusion:**

This study will ensure the extent and consequences of LC-ILD are established and enable strategies to mitigate progression of LC-ILD.

## Introduction

The COVID-19 pandemic has led to over 100 million cases worldwide. In the UK alone, there have been over 4 million cases, over 400 000 hospital admissions and over 100 000 deaths. A large number of people diagnosed with COVID-19 suffer from long-term symptoms, predominantly breathlessness and fatigue whether or not they were admitted to hospital. However, long-term symptoms following COVID-19 are more common in people who suffered more severe acute disease.^1–4^ There are a number of potential causes of long-term breathlessness following COVID-19 including thromboembolic disease, myocarditis or pericarditis and physical deconditioning. However, based on early data from the COVID-19 pandemic, and from other viral infections, a potentially severe long-term consequence of COVID-19 is the development of long COVID-related interstitial lung disease (LC-ILD).^5–7^

SARS-CoV-2 leads to pronounced inflammation within the lung and leads to the development of acute respiratory distress syndrome in a substantial proportion of those infected. While data demonstrate that a short course of corticosteroids can improve survival in patients with hypoxia, there is considerable evidence of long-term inflammation even following short-term corticosteroid therapy.^8^ While the inflammatory potential of SARS-CoV-2 is well described, the fibrogenic potential of SARS-CoV-2 is currently unknown but is predicted to be substantial based on the experience of previous COVID-19 outbreaks and emerging data from this pandemic.^3 7 9^ Meta-analysis of initial observations has identified substantial levels of LC-ILD with an estimated 27% of CT scanned patients having fibrotic changes during hospitalisation, and an estimated 33% with fibrotic changes at 6 months, which suggests minimal regression over time.^7^ Similarly, an estimated 21% of patients had lung function impairment consistent with restrictive disease and 45% had reduced gas transfer, when sampled between 1 and 6 months following COVID-19 hospitalisation.^7^ Risk factors for severe COVID-19 include increasing age, male sex and comorbidities including hypertension and type 2 diabetes mellitus,^10 11^ which are also associated with progressive lung fibrosis.^10–12^

Pilot data from our consortium^13^ indicate that novel functional imaging methods using ^129^Xe and ^1^H MRI are sensitive to gas transfer limitation, microstructural airway changes and alveolar perfusion deficit in patients with both acute and long COVID-19. These methods also have some ability to dissect both inflammatory and fibrotic pathophysiology and are highly sensitive to disease progression in established ILDs.^14–18^

Given the large number of patients infected with SARS-CoV-2, it is vital that the extent of LC-ILD is determined; its natural history defined particularly whether it is time-limited inflammation and reversible, or develops into persistent, or even progressive, fibrosis. Determining the natural history of LC-ILD, and risk factors as well as biomarkers related to outcome such as disease progression, will enable a precise approach to possible treatments such as immunomodulation or antifibrotic therapy,^6^ stratification into clinical trials, prognostication and appropriate service provision. This will facilitate assessment and prioritisation of both conventional and novel therapies used in the treatment of COVID-19 during the acute phase to mitigate the subsequent development of LC-ILD. By exploring the long-term implications of SARS-CoV-2 infection across the full spectrum of COVID-19 disease ranging from non-hospitalised patients managed in the community with mild symptoms to those requiring mechanical ventilation, we will define the risk factors for LC-ILD including disease severity, host genetic factors and the effects of antiviral and immunomodulatory treatment administered during the acute phase of the illness.

To address the development of ILD following SARS-COV-2 infection, we will determine the prevalence and natural history of LC-ILD in the 12 months following COVID-19 through linkage to the Post-Hospitalisation COVID (PHOSP-COVID) platform study, the REACT community study and unique collections such as the Xenon MRI investigation of Alveolar dysfunction Substudy (XMAS) and POST COVID-19 interstitial lung DiseasE (POSTCODE) substudies. The UK Interstitial Lung Disease Consortium (UKILD) will undertake a longitudinal observational study of patients with suspected ILD following COVID-19, to determine the clinical, genetic, epigenetic and biochemical factors that determine the trajectory of recovery or progression.

## Methods and analysis

**Figure 1:**
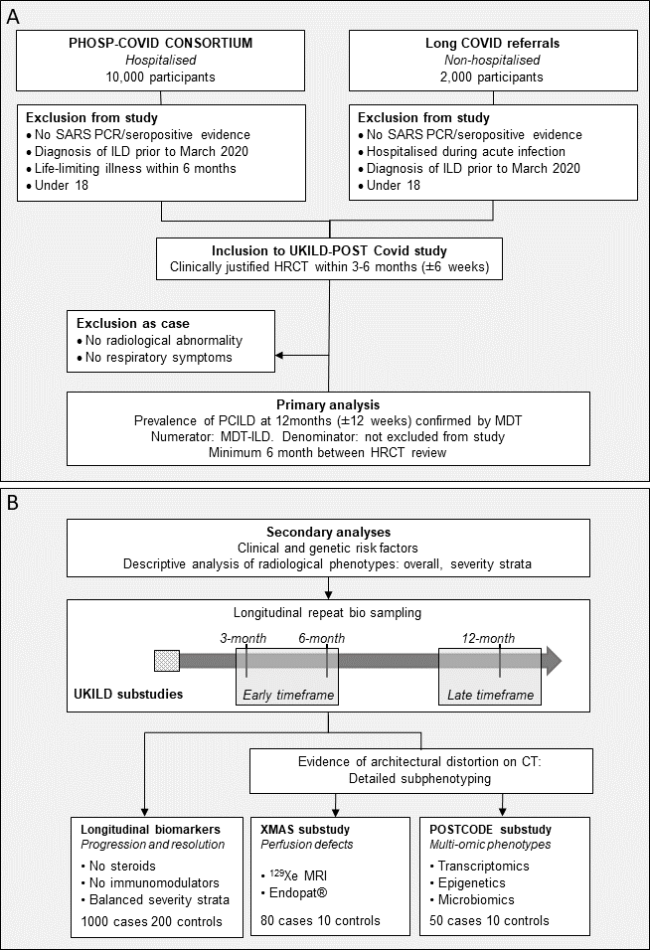
UK Interstitial Lung Disease (UKILD)-Long COVID study flow. (A) Inclusion and exclusion criteria for UKILD-Long COVID study and planned primary analysis. (B) Planned secondary analysis flow and design including UKILD-Long COVID substudies. HRCT, High Resolution Computerised Tomography; MDT, Multi-Disciplinary Team; PHOSP-COVID, Post-Hospitalisation COVID; POSTCODE, POST COVID-19 interstitial lung DiseasE; COVID ILD, Post COVID ILD; XMAS, Xenon MRI investigation of Alveolar dysfunction Substudy.

The UKILD-Long COVID study is a prospective multicentre observational cohort study that will be managed through the Imperial College National Heart and Lung Institute and funded by the UKRI Medical Research Council and an NIHR professorship (RGJ). The primary study aims to define the prevalence and risk factors for LC-ILD using data from 10 000 stratified patients, collected from the UK’s national PHOSP for COVID platform study, together with 2000 non-hospitalised patients referred for assessment up to 3 months for investigation of persistent respiratory symptoms post-COVID-19, such as breathlessness and cough (figure 1A). These patients will be recruited from Long COVID clinics or community Post-COVID-19 clinical trials (REACT Long COVID Study^19^). We will assess the clinical and genetic risk factors for post-COVID-19 ILD and radiological classification, as well as the trajectory and pathogenic mechanisms of progression following acute COVID with longitudinal analysis of biomarkers and the undertaking of two substudies (figure 1B).

1. Up to 80 participants will undergo detailed longitudinal xenon and proton MRI coupled with measures of flow-mediated endothelial function in the microvasculature measured by EndoPat.
2. Up to 50 participants will undergo bronchoscopy with bronchoalveolar lavage (BAL) for single cell and/or bulk RNA sequencing (RNA-seq), with matched flow cytometry analysis, as well as microbiome analysis.

### Objectives

The primary objective of the study is to determine the prevalence of ILD at 12 months following SARS-CoV-2 infection and whether clinical severity correlates with severity of ILD in survivors.

#### Secondary objectives

1. To further define the LC-ILD population, in particular, to describe the emerging phenotypes and risk factors of LC-ILD.
2. To determine the natural history of LC-ILD phenotypes longitudinally.
3. To explore pathomechanisms of LC-ILD for candidate prognostic and theranostic biomarkers.

#### The primary end point

The primary end point of the study is as radiologically confirmed diagnosis of fibrotic or non-fibrotic ILD in the 12 months following COVID-19. Substudies will have a co-primary end point of change in the radiological extent of LC-ILD.

#### The secondary end points

1. Progressive lung function impairment between 3 and 12 months, defined as ≥10% relative decline in Forced Vital Capacity (FVC), or ≥10% relative decline in Diffusion Capacity in Lung of Carbon Monoxide (DLco), or increasing radiological extent of LC-ILD using image analysis.^20^
2. Resolution of ILD, as defined by ≥10% relative improvement in FVC, DLco or reduction of radiological extent.
3. Persistence of ILD in those not meeting definition of progression or resolution.
4. Presence of interstitial lung abnormalities on radiological images that do not meet definition of ILD.
5. A comprehensive series of clinical, molecular, MRI and biochemical parameters will be assessed as biomarkers.

### Selection of participants

The PHOSP-COVID study (ISRCTN10980107) is a national consortium that provides a platform to study the long-term consequences of COVID hospitalisations.^21^ An expected 10 000 individuals hospitalised by COVID are to be included in baseline assessments after testing positive on PCR for SARS-C0V-2 and will be recruited through the PHOSP platform and will permit a 3-month look back period to ensure patient recruitment targets are met. The study will also include a further 2000 individuals, with proven COVID, who were not hospitalised but presented to Long COVID clinics with persistent respiratory symptoms such as breathlessness or cough and are referred for cross-sectional imaging (CT) at baseline (3 months±6 weeks after their first COVID symptoms).^22^ A total of up to 12 000 people will be assessed for longitudinal follow-up into the UKILD-Long COVID study. Following assessment of patients with Long COVID at baseline (~3–6 months postinfection as defined above), those with clinical and radiological features suggestive of ILD will be included into the UKILD-Long COVID study population. Where there are contraindications for CT and if clinically indicated, participants will be eligible for a research-guided three-dimensional ultrashort echo time (UTE) proton MRI as a surrogate for CT. Where individuals meet criteria for initial study inclusion (COVID and clinical indication for CT scanning) but have no clinical, radiological or physiological features of ILD, they will be invited to enrol as part of the control cohort for follow-up.

#### UKILD-Long COVID inclusion criteria

Patients aged ≥18 years with (a) evidence of SARS-CoV-2 infection earlier confirmed by microbiology or serology and (b) clinical suspicion of Long COVID ILD warranting CT or consideration for proton MRI.

#### POST COvid-19interstitial lung DiseasE (POSTCODE) substudy

Up to 50 participants from various severity strata including hospitalised and non-hospitalised patients will undergo clinically indicated BAL with matched whole blood sampling up to 6 months and then again between 12 months±3 months following infection with a minimum of 6 months between bronchoscopy and whole blood sampling for deep phenotyping. Assessments will include exploratory analysis such as single cell and bulk RNAseq, flow cytometry analysis, lung microbiomics and epigenetic assessment. Participants eligible for secondary analyses will be recorded and monitored on a real-time study dashboard to support balanced recruitment of gender, age and severity of acute infection. Participants in secondary analyses will be largely recruited from specialist centres for radiological imaging and bronchoscopy procedures, with other centres referring.

Patients aged ≥18 years with (a) evidence of SARS-CoV-2 infection confirmed by microbiology or serology and (b) clinical suspicion of Long COVID ILD warranting CT or consideration for proton MRI, with architectural distortion of the lung defined by abnormal displacement of bronchi, vessels, fissures or septa caused by diffuse or localised lung disease, particularly interstitial fibrosis.

#### UKILD - POSTCODE exclusion criteria

- Confirmed ILD diagnosis prior to the diagnosis of COVID-19.
- No CT chest evidence of architectural distortion.
- Life-limiting illness within 12 months.

Further exclusion criteria have been selected to support distinction of cases and controls during follow-up. Cases will have evidence of a radiological abnormality at baseline scan (up to 6 months) post-COVID-19, with architectural distortion of the lung defined by abnormal displacement of bronchi, vessels, fissures or septa caused by diffuse or localised lung disease, particularly interstitial fibrosis,^23^ while participants included in the control population will meet inclusion criteria but have no evidence of lung architectural distortion on CT or MRI as defined by local PI.^24^ No evidence of breathlessness or respiratory symptoms will act as additional criteria to select controls from the same population for comparison in longitudinal study. Spirometry and gas transfer do not contribute to essential criteria due to potential missingness regarding safety risks of aerosolisation. Specific radiological patterns will not be part of essential criteria in order to minimise selection bias and support detailed assessment of the potential range of radiological changes observed in the lung. Contraindications to MRI (ferrous implants, claustrophobia and gadolinium contrast agent risk) will be exclusion criteria for the xenon MRI substudy.

### Study flow/Regimen

Individuals meeting the inclusion criteria for UKILD-Long COVID will be followed up using the PHOSP-COVID platform, or using specific substudy consent. Clinical, molecular, biochemical and patient-reported outcome measure (PROM) data will be captured in the PHOSP platform using predefined case report forms with linkage to retrospective and prospective social and healthcare records. Up to 40% of individuals enrolled in PHOSP will undergo research-specific biosampling and linkages to enhanced clinical records. PHOSP case report forms will be completed during the acute COVID admission and at 3, 6 and 12 months posthospitalisation. Eligible non-hospitalised individuals presenting at Long COVID clinics will have physiological tests and biosampling (including whole blood plasma, serum, DNA and peripheral blood monocytes) performed alongside a full clinical history with clinical evaluation and testing. Longitudinal follow-up will be performed at least 6 months following initial assessment up to 15 months postinfection and will be guided by clinical need. The primary analysis will focus on the prevalence of MDT-confirmed ILD at any time point up to date of censorship within the wider cohort, as well as prevalence of genetic risk factors and radiological patterns, assessed overall and according to severity strata.

Participants and controls will also be eligible for enrolment into two specific predefined substudies.

#### The Xenon MRI investigation of Alveolar dysfunction Study

An anticipated 80 participants representing hospitalised patients from various severity strata including non-hospitalised patients will undergo longitudinal xenon alveolar gas transfer and proton perfusion MRI up to 6 months and then again between 12 months±3 months following infection with a minimum of 6 months between MRI scans. Pilot data from our consortium indicate that novel functional imaging methods using ^129^Xe and ^1^H MRI are sensitive to gas transfer limitation, microstructural airway changes and alveolar perfusion deficit in patients with both acute and long COVID-19. These methods also have some ability to dissect both inflammatory and fibrotic pathophysiology and are highly sensitive to disease progression in established ILDs.^14–18^ Dynamic contrast-enhanced perfusion MRI has sensitivity to regional blood volume (as does Single-Photon Emission Computerised Tomography (SPECT)) and has added dynamic sensitivity to quantitative regional blood flow, pulmonary vascular transit time and endothelial leakage all of which are of interest in understanding the accompanying endothelial and microvascular pathophysiology in COVID-ILD. The XMAS study will be performed under site-specific protocols already in place for scanning patients with acute and chronic COVID-19 in Sheffield, Nottingham and Oxford (ClinicalTrials.gov NCT04872309).

In a subset of patients, MRI will be coupled with Endopat measurements, a Food and Drug Administration-approved non-invasive tool which measures changes in flow-mediated endothelial function in the microvasculature and is sensitive to changes in systemic vascular function patients with chronic lung disease,^25 26^ to explore defects in alveolar dysfunction.

### Statistical analysis

The prevalence of MDT-confirmed LC-ILD at both early (up to 6 months) and then again at late (12 months±3 months) time points will be assessed within the total study population. The prevalence of broader radiological abnormalities and phenotypic patterns will also be assessed in a descriptive analysis, together with demographics, haematological and biochemical profiles, physiological performance and PROM. Analyses will be performed overall and stratified according to hospitalised and non-hospitalised, as well as severity of infection in hospitalised patients defined above.

**Figure 2:**
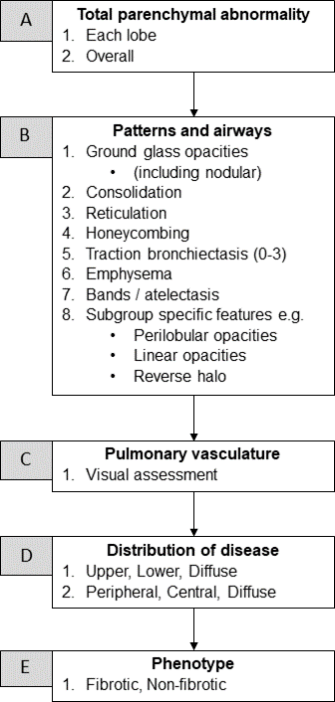
UK Interstitial Lung Disease-Long COVID radiological assessment. Classification criteria for radiological assessment of HRCT images.

All posthospitalised study participants that are part of the PHOSP-COVID study will be linked to the centralised National COVID-19 Chest Imaging Database (NCCID). Imaging data for the relevant NCCID patients will be accessible to UKILD-Long COVID study research groups after completion and approval of NCCID Data Access Requests. Similarly, imaging data from the PHOSP-COVID study will be accessible to UKILD-Long COVID study research groups after completion and approval of PHOSP-COVID Data Access Requests. NCCID will provide pre-COVID-19 and acute COVID-19 (inpatient chest X-ray (CXR) and CT) imaging, while PHOSP-COVID will provide posthospital discharge (CXR and CT) imaging for hospitalised patients only. Imaging data-flows vary slightly for NCCID and PHOSP-COVID.^27^ A link to the NCCID Github page is included in references.^28^ Each CT will be assessed according to the pipeline defined in figure 2.

For non-hospitalised patients, the imaging data transfer process will replicate that being performed in NCCID and PHOSP-COVID. Data will be sent via Image Exchange Portal (IEP) from each hospital site to Royal Surrey University Hospital NHS Foundation Trust, and then to the National Consortium of Intelligent Medical Imaging (NCIMI) for storage and distribution.

Putative genetic risk factors for lung fibrosis or severe COVID-19 outcomes, as defined by genome-wide association screens,^29 30^ as well as treatments (eg, antivirals, immunomodulation), comorbidities and initial disease severity, will be tested for their association with LC-ILD and radiological progression, continuously and dichotomised. Risk factors will be assessed in generalised linear models, performed overall and according to major radiological patterning phenotypes. Models will be univariable and adjusted for a priori defined characteristics of age, gender, body mass index, smoking history. Directed-acyclic graphs will be specified to ensure the minimum adjustments are included when estimating associations of risk factors. To identify valuable predictors in exploratory analysis of a large number of reported or measured features, variable selection techniques such as least angle regression with bootstrapping,^31^ and partial least squares will be used. A complete case analysis will be performed with missingness in data fields reported.

Baseline and longitudinal changes in biomarkers reflecting LC-ILD evolution, including circulating factors and cell-types from detailed serological and cellular analysis, will be assessed in multilevel models for repeated measures to test associations according to the presence or absence of LC-ILD at late time points (12 months±3 months), and according to progression, resolution or persistence of radiological patterns over follow-up time points.

A restricted sample of participants with presence of architectural distortion confirmed by HRCT will undergo deep phenotyping in the two separate substudies XMAS and POSTCODE.

XMAS will capture radiological changes in longitudinal ^129^hyperpolarised xenon MRI (Xe-MR) coupled with measures of endothelial dysfunction (EndoPAT) between early and late time points. Paired analyses will be performed to test the hypothesis that the red blood cell to tissue plasma ratio (RBC/TP), or diffusion-weighted mean diffusive length scale (LmD), measured by Xe-MR, reduces over time in people with LC-ILD; alveolar-interstitial-capillary gas transfer (RBC/TP), acinar microstructure (LmD) and Dynamic Contrast Enhanced (DCE) lung perfusion (mean capillary transit time (MTT), pulmonary blood volume (PBV)) have been observed to be sensitive to progression in IPF.^14–18^ Changes in Xe-MR indices will be modelled longitudinally and tested for correlations with changes in lung function indices, FVC and DLco, biomarkers of endothelial-derived vasoactive/thrombotic hormones as well as systemic vascular function. Regional maps of MRI functional parameters will be registered to ^1^H and CT structural imaging and calliper. Image analysis from data acquired at the three-^129^XE MRI sites will be performed centrally in Sheffield with input from UCL on the quantitative CT, Oxford and Nottingham (free breathing ^1^H MRI).

The primary analysis will be the change in radiological extent between early and late time points as measured by ^129^Xe RBC/TP in paired analysis. Secondary analyses will assess changes in ^129^Xe LmD, and ^1^H DCE MTT, PBV, as well as differences between severity strata with longitudinal modelling data.

POSTCODE will perform multi-omic phenotyping at early time points (up to 6 months) and assess potential changes later time points (12 months±3 months). Single cell RNA-seq and lung microbiome^32^ analysis will be performed on a proportion of the samples obtained from clinical BAL up to 6 months following acute infection to determine associations with LC-ILD.^33^ Participants will also be offered to consent for a second bronchoscopy at 12 months±3 months to enable paired analysis. Fibrogenic responses will be explored at single cell resolution, and validated by flow cytometry, and the role of the microbial community explored. Centres will perform procedures and analyses using standardised protocols and bioinformatic pipelines.

In addition, this cohort will undergo bulk whole blood RNAseq and analysis of epigenetic modifications at both the early and late time points to identify longitudinal biological signatures reflecting distinct resolving phenotypes, compared with persisting or progressive. Defining epigenetic signatures will test the hypothesis that COVID-19 promotes epigenetic changes that can predispose people to lung fibrosis.

The primary aim is to characterise the genetic and epigenetic signal from the airways and the microbiome in patients with pulmonary fibrosis post-SARS-Cov-2 infection, while secondary aims will be to elucidate changes in the host and microbiome over time following SARS-Cov-2 infection, as well as differences in the microbiome, genetic and epigenetic signatures between patients with progressive and non-progressive disease.

Primary analyses will assess the change in the multi-omic signals between early and late time points in paired analysis according to the separate platforms. Secondary analyses will determine biological signatures of resolving LC-ILD phenotypes compared with non-resolving in baseline and longitudinal models.

### Sample size justification

#### UKILD-Long COVID

Up to 10 000 participants are expected to be recruited through the PHOSP-COVID consortium, with a further 2000 non-hospitalised participants recruited. Post-COVID-related breathlessness has been reported to affect approximately 40% of people hospitalised with severe disease and 10%–20% of people who suffered mild COVID. Preliminary analysis from UK and international studies have described up to 80% of hospitalised patients with COVID-19 have the presence of symptoms, radiology and lung function changes concordant with ILD, depending on the severity of the underlying illness. We anticipate approximately 20% (2400/12 000) of people recruited into UKILD-Long COVID study will have features consistent with ILD for characterisation, which will provide sufficient sample size for descriptive analysis, evaluation of incidence and prevalence and assessment of risk factors.

Genome-wide genotype data are funded through GenOMICC and will be available for >4000 participants enrolled in PHOSP-COVID; we will conduct genome-wide array genotyping in an additional 2000 non-hospitalised participants. A common MUC5B polymorphism associated with (non-COVID) idiopathic pulmonary fibrosis has an OR for disease >2. Anticipating approximately 20% of non-hospitalised participants will have features consistent with ILD (400/2000), there will be sufficient power to detect associations with ORs >1.65 (minor allele frequency 11%) at p<0.0025 with correction for multiple testing of a restricted set of 20 independent variants.

An anticipated 1000 LC-ILD participants representing balanced strata of initial severity and a minimum 200 controls will be assessed in longitudinal analysis to identify biomarkers that describe the natural history. Data from prior biomarker studies in IPF estimate a sample size of 1000 achieves 90% power to detect a 13 ng/mL difference in mean MMP7 levels, with a combined SD of 0.18 ng/mL in an unbalanced analysis assuming 85% resolving disease, providing a standardised effect of 0.72.^34^ To minimise confounding in more restricted samples sizes, analyses will exclude individuals receiving steroids or immunomodulatory therapy at baseline assessment.

#### Xenon MRI investigation of Alveolar dysfunction Substudy

The Xe-MR substudy will be staged. Stage 1 will include an interim analysis of the effect and variability between 12-month (first wave) and 3-month (second wave) unpaired scans after recruitment of 20 participants to each arm. Only hospitalised participants with radiological evidence of architectural distortion will be enrolled at stage 1. Stage 2 will use the findings from stage 1 to guide strata representation and required sample size for a longitudinal analysis of Xe-MR, with an anticipated maximum of 80 cases imaged at both early and late time points. A sample size of 80 repeat scans will provide 90% power to observe effect sizes above 0.40 at alpha 0.05, assuming more variability in LC-ILD than observed in IPF.

#### POST COVID-19 interstitial lung DiseasE

The BAL substudy will be performed in up to 50 cases representing balanced numbers of hospitalised and non-hospitalised participants, and 10 controls. Participants will be invited to support the strength of these data through a follow-up bronchoscopy at 12 (12 months±3 months) months and repeat measures. Participants who undergo bronchoscopy will be consented for whole blood sampling to perform longitudinal whole blood RNA-seq and epigenetic analyses. Assuming 50 cases, medium-sized effects in exploratory biological signatures (Cohen’s d=0.5) will be powered at >90% in paired analysis with no correction for familywise error, or 70% when controlling for 15 independent comparisons. Large effects (Cohen’s d=0.8) will be powered at a similar rate when controlling for 10 000 comparisons.

#### Planned interim analyses

As management of Long COVID and post-COVID-19 pathologies must be adaptive and responsive to novel research findings, two planned interim analyses are to be conducted to inform the likely number of outcome events and the expected power in substudies. The first planned interim will be performed after 3-month follow-up of the first 1000 participants through PHOSP (10%) to provide initial insights into expected radiological patterns and to preliminarily describe the potential LC-ILD burden for the support of clinical management. The second planned interim will be performed following completion of 20 12-month Xe-MR from the primary wave of infection and 20 3-month Xe-MR from secondary waves. An unpaired analysis will assess the difference, variability and standardised effect to inform recruitment to the longitudinal Xe-MR cohort.

### Ethics and dissemination

#### Study monitoring

- Data verification, case report forms (CRFs), study co-ordinator.

This study will be conducted in accordance with the International Conference on Harmonisation of Good Clinical Practice (ICH GCP), the research governance framework for health and social care and according to the principles as outlined in the Declaration of Helsinki.

All conduct within the study will comply with the principles outlined in the Declaration of Helsinki and GCP. A study coordinator will ensure compliance with good clinical practice, support collaborative working with independent partners (PHOSP consortium, local clinics, industry and regulatory bodies), oversee central recruitment targets for substudies and support public dissemination.

Electronic CRFs will be used to collect data. Data will be submitted electronically to a protected online database. Anonymised data may be entered by study staff in order to minimise workload on site clinical staff. Patient identities will be protected and all information held securely. Sharing of data with industrial partners will comply with General Data Protection Regulations.

Data entered on CRFs and the online database will be subject to quality checks to reference source data on a regular basis by the study co-ordinator or nominated deputy to ensure standardisation and validity of the data collected. The study may be monitored by the sponsor or other regulatory bodies. For the purposes of audit and compliance monitoring, clinical study data will be available to delegated members of the local study teams, as well as to representatives of the sponsor and the study co-ordinating team. All trial data, data monitoring and audit will be available for inspection as necessitated by research ethics committee inspection. Participants will be consented to their study data being released for this purpose.

#### Safety reporting

All of the investigations we are performing as part of this research are safe and happen in the hospital on a daily basis. However, as with all procedures there are risks of complications. Patients are at risk of a small amount of discomfort from the blood tests required as part of the study. Venepuncture will be performed by qualified personnel according to best practice techniques to minimise any adverse events (AEs) and where possible, taken at the same time as clinically indicated blood samples to avoid repeated sampling.

For patients undergoing bronchoscopy, the possible risks are detailed in the POSTCODE patient information sheet. Common side effects are a transient sore throat and hoarse voice and fever like influenza symptoms can occur and last for 24–48 hours and usually settle spontaneously or with simple analgesia. A rare complication is a chest infection, this occurs in <1% of cases.^35^

For patients undergoing Xenon or proton MRI, the possible risks are detailed in the respective patient information sheets. Ethics for the XMAS substudy are in place at the respective sites of Sheffield, Nottingham and Oxford—see accompanying protocols (Sheffield—Murdoch IRAS ethics ref 265997 19/LO/1115, folio adoption reference 42586; Oxford—C-MORE-POST IRAS ethics ref 282608 20/NW/0235; Nottingham—UKILD-Long COVID IRAS ref 19/NE/0330).

All AEs will be recorded and monitored until resolution or until it has determined that intervention as part of the study was not the cause. All AEs, whether expected or not, will be recorded. All serious adverse events (SAEs) will be reported to the Chief Investigator within 24 hours. However, hospitalisations for elective treatment of a pre-existing condition do not need reporting as SAEs.

#### Study oversight

The trial will be overseen by the UKILD consortium steering committee. The first planned interim analysis will be performed after 3-month follow-up of the first 1000 hospitalised participants, recruited through PHOSP-COVID, to provide initial insights into expected radiological patterns and the potential LC-ILD burden. The second planned interim will be performed following completion of 20 independent 12-month Xe-MR scans from the primary wave of infection and 20 independent 3-month Xe-MR scans from secondary waves.

#### Patient and public involvement

Action for pulmonary fibrosis charity and patient partners have been involved from the conception of this study to inform study design and conduct. They will also form part of the ongoing steering committee appraising patient facing material and facilitating the dissemination of findings to IPF patients and their families.

#### Dissemination plan

The results from this study will be disseminated via regional and national conference platforms, as well as being submitted for publication in open access peer-reviewed journals in accordance with UK Research Council policies. Any publication will include a list of investigators, with authors being determined in line with the International Committee of Medical Journal Editors guidelines, as well as an acknowledgement of roles of the study sponsor and funder(s).

## Conclusion

This longitudinal observational study was designed by the UKILD consortium in response to the global SARS-CoV-2 pandemic to proactively characterise viral-induced lung changes. UKILD represents a network of national ILD centres with integrated academic groups working in collaboration with large platforms for COVID-19 research, including the Coronavirus Clinical Characterisation Consortium (ISARIC-4C), PHOSP-COVID platform and REACT Long COVID studies. The study will inform the prevalence and burden of LC-ILD to improve local referral pathways and guide follow-up where fibrosis is suspected, while findings regarding the natural history and mechanisms of LC-ILD will support discovery of diagnostic, prognostic and theranostic biomarkers to improve patient management. Collectively, the objectives of this study will ensure that the extent and consequences of LC-ILD are established using existing and novel diagnostic approaches, informing the design of future clinical trials and strategies to mitigate disease progression.
